# *N*′-[1-(4-Chloro­phen­yl)ethyl­idene]benzo­hydrazide

**DOI:** 10.1107/S1600536811042887

**Published:** 2011-10-29

**Authors:** Suyuan Zeng, Lei Li

**Affiliations:** a College of Chemistry and Chemical Engineering, Liaocheng University, Shandong 252059, People’s Republic of China

## Abstract

In the title mol­ecule, C_15_H_13_ClN_2_O, the two benzene rings form a dihedral angle of 5.48 (4)°. In the crystal, N—H⋯O hydrogen bonds link mol­ecules related by translation along the *a* axis into chains, which are further aggregated into layers parallel to the *ac* plane through weak C—H⋯O and C—H⋯N inter­actions.

## Related literature

For applications of Schiff base derivatives and their complexes, see: Chavan *et al.* (2011 ▶); Ray *et al.* (2011 ▶). For the crystal structures of related compounds, see: Nie (2008 ▶); Fun *et al.* (2008 ▶).
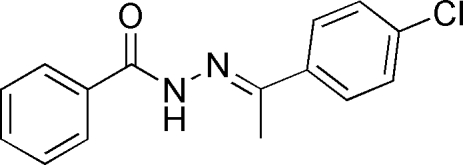


## Experimental

### 

#### Crystal data

C_15_H_13_ClN_2_O
*M* *_r_* = 272.72
Monoclinic, 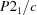

*a* = 5.0714 (6) Å
*b* = 31.430 (3) Å
*c* = 8.4128 (7) Å
β = 94.388 (1)°
*V* = 1337.0 (2) Å^3^
*Z* = 4
Mo *K*α radiation
μ = 0.28 mm^−1^
*T* = 298 K
0.40 × 0.30 × 0.12 mm

#### Data collection

Bruker SMART APEX CCD area-etector diffractometer
Absorption correction: multi-scan (*SADABS*; Sheldrick, 1996 ▶) *T*_min_ = 0.897, *T*_max_ = 0.967
6717 measured reflections
2322 independent reflections
711 reflections with *I* > 2σ(*I*)
*R*_int_ = 0.156

#### Refinement

*R*[*F* ^2^ > 2σ(*F* ^2^)] = 0.089
*wR*(*F* ^2^) = 0.205
*S* = 1.01
2322 reflections
173 parameters
H-atom parameters constrained
Δρ_max_ = 0.31 e Å^−3^
Δρ_min_ = −0.24 e Å^−3^

### 

Data collection: *SMART* (Bruker, 2007 ▶); cell refinement: *SAINT* (Bruker, 2007 ▶); data reduction: *SAINT*; program(s) used to solve structure: *SHELXS97* (Sheldrick, 2008 ▶); program(s) used to refine structure: *SHELXL97* (Sheldrick, 2008 ▶); molecular graphics: *SHELXTL* (Sheldrick, 2008 ▶); software used to prepare material for publication: *SHELXTL*.

## Supplementary Material

Crystal structure: contains datablock(s) I, global. DOI: 10.1107/S1600536811042887/cv5164sup1.cif

Structure factors: contains datablock(s) I. DOI: 10.1107/S1600536811042887/cv5164Isup2.hkl

Supplementary material file. DOI: 10.1107/S1600536811042887/cv5164Isup3.cml

Additional supplementary materials:  crystallographic information; 3D view; checkCIF report

## Figures and Tables

**Table 1:** Hydrogen-bond geometry (Å, °)

*D*—H⋯*A*	*D*—H	H⋯*A*	*D*⋯*A*	*D*—H⋯*A*
N1—H1⋯O1^i^	0.86	2.32	3.067 (6)	145
C9—H9*C*⋯N2^i^	0.96	2.53	3.452 (8)	162
C15—H15⋯O1^ii^	0.93	2.61	3.483 (7)	157

Symmetry codes: (i) 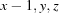; (ii) 
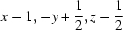
.

**Fig. 1.**
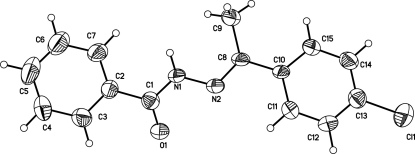
View of (I) showing the atomic numbering and 30% probability displacement ellipsoids.

## supplementary crystallographic information

### Comment

Schiff bases have various applications in the study of biological processes and in pharmacology (Chavan *et al.*, 2011; Ray *et al.*, 2011). We report here the crystal structure of the title Schiff base compound (I).

In (I) (Fig. 1), the bond lengths and angles are normal and comparable to the values observed in similar compounds (Nie, 2008; Fun *et al.*, 2008). The C═N (C8═N2) bond length in the molecule is 1.270 (7) Å showing the double-bond character. Meanwhile, the dihedral angle between the benzene rings C2–C7 and C10–C15 is 5.48 (4)°.

In the crystal stucture, intermolecular N—H···O hydrogen bonds (Table 1) link the molecules related by translation along axis *a* into chains, which are further aggregated into layers parallel to *ac* plane through the weak C—H···O(N) interactions (Table 1).

### Experimental

Benzohydrazide (5.0 mmol), 20 ml ethanol and 4-chloroacetophenone (5.0 mmol) were mixed in 50 ml flash. After refluxing 3 h, the resulting mixture was cooled to room temperature, and recrystalized from ethanol, and afforded the title compound as a crystalline solid.

### Refinement

All H atoms were placed in geometrically idealized positions (N—H 0.86 and C—H 0.93–0.96 Å) and treated as riding on their parent atoms, with *U*_iso_(H) = 1.2–1.5*U*_eq_(C,N).

### Crystal data

**Table:**

C_15_H_13_ClN_2_O	*F*(000) = 568
*M**_r_* = 272.72	*D*_x_ = 1.355 Mg m^−^^3^
Monoclinic, *P*2_1_/*c*	Mo *K*α radiation, λ = 0.71073 Å
*a* = 5.0714 (6) Å	Cell parameters from 385 reflections
*b* = 31.430 (3) Å	θ = 2.6–18.2°
*c* = 8.4128 (7) Å	µ = 0.28 mm^−^^1^
β = 94.388 (1)°	*T* = 298 K
*V* = 1337.0 (2) Å^3^	Needle, colourless
*Z* = 4	0.40 × 0.30 × 0.12 mm

### Data collection

**Table:**

Bruker SMART APEX CCD area-etector diffractometer	2322 independent reflections
Radiation source: fine-focus sealed tube	711 reflections with *I* > 2σ(*I*)
graphite	*R*_int_ = 0.156
φ and ω scans	θ_max_ = 25.0°, θ_min_ = 2.6°
Absorption correction: multi-scan (*SADABS*; Sheldrick, 1996)	*h* = −5→6
*T*_min_ = 0.897, *T*_max_ = 0.967	*k* = −36→37
6717 measured reflections	*l* = −9→9

### Refinement

**Table:**

Refinement on *F*^2^	Primary atom site location: structure-invariant direct methods
Least-squares matrix: full	Secondary atom site location: difference Fourier map
*R*[*F*^2^ > 2σ(*F*^2^)] = 0.089	Hydrogen site location: inferred from neighbouring sites
*wR*(*F*^2^) = 0.205	H-atom parameters constrained
*S* = 1.01	*w* = 1/[σ^2^(*F*_o_^2^) + (0.0367*P*)^2^] where *P* = (*F*_o_^2^ + 2*F*_c_^2^)/3
2322 reflections	(Δ/σ)_max_ < 0.001
173 parameters	Δρ_max_ = 0.31 e Å^−^^3^
0 restraints	Δρ_min_ = −0.24 e Å^−^^3^

### Special details

**Table:**

Geometry. All e.s.d.'s (except the e.s.d. in the dihedral angle between two l.s. planes) are estimated using the full covariance matrix. The cell e.s.d.'s are taken into account individually in the estimation of e.s.d.'s in distances, angles and torsion angles; correlations between e.s.d.'s in cell parameters are only used when they are defined by crystal symmetry. An approximate (isotropic) treatment of cell e.s.d.'s is used for estimating e.s.d.'s involving l.s. planes.
Refinement. Refinement of *F*^2^ against ALL reflections. The weighted *R*-factor *wR* and goodness of fit *S* are based on *F*^2^, conventional *R*-factors *R* are based on *F*, with *F* set to zero for negative *F*^2^. The threshold expression of *F*^2^ > σ(*F*^2^) is used only for calculating *R*-factors(gt) *etc*. and is not relevant to the choice of reflections for refinement. *R*-factors based on *F*^2^ are statistically about twice as large as those based on *F*, and *R*-factors based on ALL data will be even larger.

### Fractional atomic coordinates and isotropic or equivalent isotropic displacement parameters (Å^2^)

**Table:**

	*x*	*y*	*z*	*U*_iso_*/*U*_eq_	
N1	0.1612 (9)	0.19148 (16)	0.2256 (5)	0.0650 (15)	
H1	0.0012	0.1841	0.1975	0.078*	
N2	0.2278 (10)	0.23422 (17)	0.2404 (6)	0.0623 (14)	
O1	0.5839 (9)	0.17193 (12)	0.2838 (5)	0.0807 (14)	
Cl1	0.3944 (4)	0.44187 (5)	0.2883 (3)	0.1218 (10)	
C1	0.3466 (15)	0.1622 (2)	0.2551 (7)	0.0666 (18)	
C2	0.2677 (14)	0.11724 (19)	0.2454 (9)	0.072 (2)	
C3	0.4245 (14)	0.0876 (2)	0.3254 (8)	0.091 (2)	
H3	0.5794	0.0961	0.3833	0.109*	
C4	0.354 (2)	0.0454 (2)	0.3206 (11)	0.115 (3)	
H4	0.4591	0.0259	0.3790	0.138*	
C5	0.135 (2)	0.0313 (3)	0.2335 (14)	0.128 (4)	
H5	0.0881	0.0027	0.2317	0.154*	
C6	−0.0118 (16)	0.0606 (3)	0.1496 (12)	0.130 (3)	
H6	−0.1606	0.0520	0.0864	0.156*	
C7	0.0548 (14)	0.1034 (2)	0.1558 (9)	0.097 (3)	
H7	−0.0502	0.1228	0.0966	0.116*	
C8	0.0706 (13)	0.2614 (2)	0.1746 (8)	0.0627 (17)	
C9	−0.1756 (12)	0.24969 (16)	0.0768 (8)	0.083 (2)	
H9A	−0.1467	0.2240	0.0190	0.124*	
H9B	−0.2230	0.2722	0.0030	0.124*	
H9C	−0.3161	0.2453	0.1453	0.124*	
C10	0.1404 (12)	0.30593 (19)	0.2013 (8)	0.0571 (17)	
C11	0.3468 (14)	0.3164 (2)	0.3061 (9)	0.088 (2)	
H11	0.4385	0.2948	0.3619	0.106*	
C12	0.4259 (13)	0.3580 (2)	0.3330 (8)	0.090 (2)	
H12	0.5711	0.3639	0.4040	0.108*	
C13	0.2931 (16)	0.39022 (19)	0.2566 (9)	0.0744 (19)	
C14	0.0872 (14)	0.3808 (2)	0.1543 (9)	0.083 (2)	
H14	−0.0043	0.4027	0.0999	0.100*	
C15	0.0069 (12)	0.3392 (2)	0.1273 (7)	0.077 (2)	
H15	−0.1404	0.3337	0.0576	0.093*	

### Atomic displacement parameters (Å^2^)

**Table:**

	*U*^11^	*U*^22^	*U*^33^	*U*^12^	*U*^13^	*U*^23^
N1	0.046 (3)	0.067 (4)	0.079 (4)	0.003 (3)	−0.015 (3)	−0.008 (3)
N2	0.055 (4)	0.070 (4)	0.060 (3)	0.001 (3)	−0.010 (3)	0.001 (3)
O1	0.063 (3)	0.077 (3)	0.100 (4)	0.000 (2)	−0.009 (3)	0.002 (2)
Cl1	0.1365 (18)	0.0779 (13)	0.147 (2)	−0.0169 (12)	−0.0117 (16)	−0.0044 (13)
C1	0.062 (5)	0.072 (5)	0.066 (5)	0.001 (5)	0.007 (5)	−0.001 (4)
C2	0.051 (5)	0.060 (5)	0.106 (6)	0.002 (4)	0.007 (5)	−0.006 (4)
C3	0.099 (6)	0.081 (5)	0.088 (6)	0.012 (5)	−0.016 (5)	−0.008 (5)
C4	0.142 (9)	0.064 (6)	0.138 (8)	0.013 (6)	−0.001 (7)	0.009 (5)
C5	0.109 (8)	0.072 (6)	0.208 (12)	−0.015 (6)	0.043 (8)	−0.014 (7)
C6	0.098 (7)	0.089 (7)	0.201 (11)	−0.007 (6)	−0.003 (7)	−0.030 (7)
C7	0.056 (5)	0.078 (5)	0.155 (8)	0.011 (4)	−0.007 (5)	−0.029 (5)
C8	0.039 (4)	0.076 (5)	0.071 (5)	0.002 (4)	−0.009 (4)	−0.003 (4)
C9	0.071 (5)	0.079 (4)	0.096 (6)	−0.004 (4)	−0.009 (5)	0.014 (4)
C10	0.033 (4)	0.066 (5)	0.070 (5)	0.001 (3)	−0.002 (4)	−0.007 (4)
C11	0.087 (6)	0.061 (5)	0.112 (6)	0.011 (4)	−0.019 (5)	0.001 (4)
C12	0.085 (5)	0.087 (5)	0.092 (6)	0.005 (5)	−0.035 (5)	−0.016 (5)
C13	0.066 (5)	0.068 (5)	0.090 (5)	−0.006 (4)	0.010 (5)	0.006 (4)
C14	0.073 (5)	0.069 (5)	0.105 (6)	−0.006 (4)	−0.010 (5)	0.027 (4)
C15	0.065 (5)	0.069 (5)	0.094 (6)	0.004 (4)	−0.016 (4)	0.008 (4)

### Geometric parameters (Å, °)

**Table:**

N1—C1	1.326 (6)	C7—H7	0.9300
N1—N2	1.388 (5)	C8—C10	1.458 (7)
N1—H1	0.8600	C8—C9	1.488 (8)
N2—C8	1.266 (6)	C9—H9A	0.9600
O1—C1	1.248 (6)	C9—H9B	0.9600
Cl1—C13	1.717 (6)	C9—H9C	0.9600
C1—C2	1.468 (7)	C10—C11	1.357 (7)
C2—C7	1.341 (8)	C10—C15	1.369 (7)
C2—C3	1.368 (8)	C11—C12	1.382 (7)
C3—C4	1.374 (9)	C11—H11	0.9300
C3—H3	0.9300	C12—C13	1.351 (8)
C4—C5	1.357 (9)	C12—H12	0.9300
C4—H4	0.9300	C13—C14	1.335 (8)
C5—C6	1.350 (10)	C14—C15	1.383 (7)
C5—H5	0.9300	C14—H14	0.9300
C6—C7	1.385 (8)	C15—H15	0.9300
C6—H6	0.9300		
			
C1—N1—N2	119.4 (5)	N2—C8—C9	123.3 (6)
C1—N1—H1	120.3	C10—C8—C9	120.4 (6)
N2—N1—H1	120.3	C8—C9—H9A	109.5
C8—N2—N1	118.2 (5)	C8—C9—H9B	109.5
O1—C1—N1	121.7 (6)	H9A—C9—H9B	109.5
O1—C1—C2	120.1 (6)	C8—C9—H9C	109.5
N1—C1—C2	118.1 (6)	H9A—C9—H9C	109.5
C7—C2—C3	117.9 (6)	H9B—C9—H9C	109.5
C7—C2—C1	123.2 (7)	C11—C10—C15	116.1 (6)
C3—C2—C1	118.8 (7)	C11—C10—C8	120.0 (6)
C2—C3—C4	120.1 (7)	C15—C10—C8	123.9 (6)
C2—C3—H3	120.0	C10—C11—C12	122.4 (6)
C4—C3—H3	120.0	C10—C11—H11	118.8
C5—C4—C3	122.3 (8)	C12—C11—H11	118.8
C5—C4—H4	118.9	C13—C12—C11	120.2 (6)
C3—C4—H4	118.9	C13—C12—H12	119.9
C6—C5—C4	116.9 (9)	C11—C12—H12	119.9
C6—C5—H5	121.5	C14—C13—C12	118.6 (6)
C4—C5—H5	121.5	C14—C13—Cl1	121.3 (6)
C5—C6—C7	121.4 (9)	C12—C13—Cl1	120.1 (7)
C5—C6—H6	119.3	C13—C14—C15	121.4 (6)
C7—C6—H6	119.3	C13—C14—H14	119.3
C2—C7—C6	121.3 (7)	C15—C14—H14	119.3
C2—C7—H7	119.4	C10—C15—C14	121.2 (6)
C6—C7—H7	119.4	C10—C15—H15	119.4
N2—C8—C10	116.3 (6)	C14—C15—H15	119.4

### Hydrogen-bond geometry (Å, °)

**Table:**

*D*—H···*A*	*D*—H	H···*A*	*D*···*A*	*D*—H···*A*
N1—H1···O1^i^	0.86	2.32	3.067 (6)	145.
C9—H9C···N2^i^	0.96	2.53	3.452 (8)	162.
C15—H15···O1^ii^	0.93	2.61	3.483 (7)	157.

Symmetry codes: (i) *x*−1, *y*, *z*; (ii) *x*−1, −*y*+1/2, *z*−1/2.
